# Benzyl Isothiocyanate Causes FoxO1-Mediated Autophagic Death in Human Breast Cancer Cells

**DOI:** 10.1371/journal.pone.0032597

**Published:** 2012-03-22

**Authors:** Dong Xiao, Ajay Bommareddy, Su-Hyeong Kim, Anuradha Sehrawat, Eun-Ryeong Hahm, Shivendra V. Singh

**Affiliations:** 1 Department of Pharmacology and Chemical Biology, University of Pittsburgh School of Medicine, Pittsburgh, Pennsylvania, United States of America; 2 Department of Pharmaceutical Sciences, Wilkes University School of Pharmacy, Wilkes Barre, Pennsylvania, United States of America; 3 University of Pittsburgh Cancer Institute, University of Pittsburgh School of Medicine, Pittsburgh, Pennsylvania, United States of America; Thomas Jefferson University, United States of America

## Abstract

Benzyl isothiocyanate (BITC), a constituent of edible cruciferous vegetables, inhibits growth of breast cancer cells but the mechanisms underlying growth inhibitory effect of BITC are not fully understood. Here, we demonstrate that BITC treatment causes FoxO1-mediated autophagic death in cultured human breast cancer cells. The BITC-treated breast cancer cells (MDA-MB-231, MCF-7, MDA-MB-468, BT-474, and BRI-JM04) and MDA-MB-231 xenografts from BITC-treated mice exhibited several features characteristic of autophagy, including appearance of double-membrane vacuoles (transmission electron microscopy) and acidic vesicular organelles (acridine orange staining), cleavage of microtubule-associated protein 1 light chain 3 (LC3), and/or suppression of p62 (p62/SQSTM1 or sequestosome 1) expression. On the other hand, a normal human mammary epithelial cell line (MCF-10A) was resistant to BITC-induced autophagy. BITC-mediated inhibition of MDA-MB-231 and MCF-7 cell viability was partially but statistically significantly attenuated in the presence of autophagy inhibitors 3-methyl adenine and bafilomycin A1. Stable overexpression of Mn-superoxide dismutase, which was fully protective against apoptosis, conferred only partial protection against BITC-induced autophagy. BITC treatment decreased phosphorylation of mTOR and its downstream targets (P70s6k and 4E-BP1) in cultured MDA-MB-231 and MCF-7 cells and MDA-MB-231 xenografts, but activation of mTOR by transient overexpression of its positive regulator Rheb failed to confer protection against BITC-induced autophagy. Autophagy induction by BITC was associated with increased expression and acetylation of FoxO1. Furthermore, autophagy induction and cell growth inhibition resulting from BITC exposure were significantly attenuated by small interfering RNA knockdown of FoxO1. In conclusion, the present study provides novel insights into the molecular circuitry of BITC-induced cell death involving FoxO1-mediated autophagy.

## Introduction

Breast cancer continues to be a leading cause of cancer-related deaths in women worldwide even after remarkable progress towards targeted therapies [1]. Novel approaches for chemoprevention of breast cancer are clinically attractive because many of the known risk factors associated with this devastating disease (e.g., family history and delayed menopause) are beyond human control and currently available chemopreventive options, such as selective estrogen receptor modulators (e.g., tamoxifen) and aromatase inhibitors, are sub-optimal [2]–[5]. Previous research in our laboratory as well as by others identifies benzyl isothiocyanate (BITC), a constituent of edible cruciferous vegetables such as garden cress, as a promising chemopreventive agent against breast cancer [6]–[11]. Possible clinical application of BITC for prevention of mammary cancer is supported by the following preclinical observations: (a) BITC inhibits chemically-induced mammary cancer in Sprague-Dawley rats [6], (b) BITC inhibits growth of cultured human breast cancer cells regardless of the estrogen receptor expression or the p53 status [7]–[9], (c) growth of a triple negative human breast cancer cell line (MDA-MB-231) subcutaneously implanted in female athymic mice is significantly retarded by BITC administration [10], and (d) dietary feeding of 3 µmol BITC/g diet suppresses mammary hyperplasia and carcinoma incidence and/or burden in MMTV-*neu* transgenic mice without any signs of overt toxicity [11]. Furthermore, population-based case-control studies suggest that dietary intake of cruciferous vegetables may be protective against breast cancer [12], [13].

Even though the mechanisms underlying growth inhibitory effect of BITC against breast cancer are not fully understood, we have shown previously that BITC treatment inhibits complex III of the mitochondrial respiratory chain leading to production of reactive oxygen species (ROS), activation of c-Jun N-terminal kinase-Bax axis, and ultimately apoptotic cell death in MDA-MB-231 and MCF-7 cells [9], [14]. Molecular circuitry of BITC-induced apoptosis downstream of ROS production also involves suppression of X-linked inhibitor of apoptosis protein [15]. We showed further that while p53 tumor suppressor is dispensable for BITC-induced apoptosis, this chemopreventive agent is capable of suppressing oncogenic actions of leptin through inhibition of signal transducer and activator of transcription 3 in human breast cancer cells [15], [16].

Because ROS production is implicated in induction of autophagy [17], which is an evolutionary conserved process for bulk degradation of cellular components including organelles (e.g., mitochondria) and considered a valid cancer chemotherapeutic target [18], we raised the question of whether growth suppressive effect of BITC was associated with autophagy induction. The present study systematically addresses this question using cultured breast cancer cells (MDA-MB-231, MCF-7, MDA-MB-468, BT-474, and BRI-JM04), a spontaneously immortalized and non-tumorigenic normal human mammary epithelial cell line (MCF-10A), and MDA-MB-231 xenografts from control and BITC-treated mice as models.

## Results

### BITC Treatment Caused Autophagy in Cultured and Xenografted Human Breast Cancer Cells

**Figure 1:**
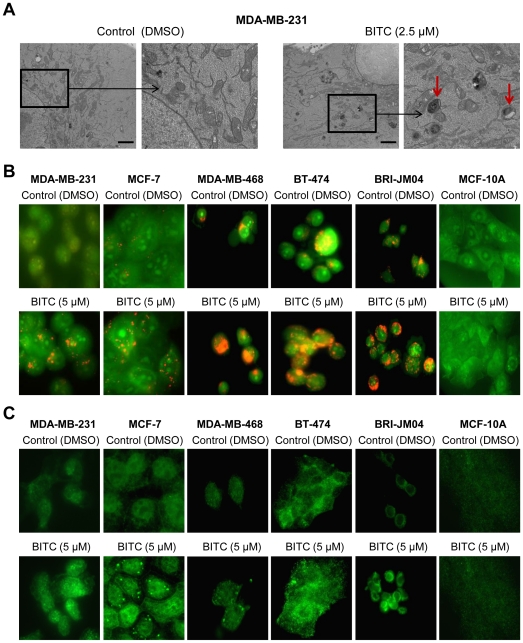
BITC treatment causes autophagy in breast cancer cells. (A) Representative transmission electron micrographs depicting ultrastructure of MDA-MB-231 cells following 12 h treatment with DMSO (control) or 2.5 µM BITC. Scale bar- 2 µm (10,000× direct magnification). Boxed area is amplified in right panel for control and BITC-treated cells for better visualization. Structures resembling autophagosomes in BITC-treated MDA-MB-231 cells are identified with red arrows. (B) Visualization of acidic vesicular organelles (yellow-orange) following acridine orange staining in DMSO-treated control and BITC-treated cells (6 h treatment except for MCF-7 cells that were treated for 9 h; objective magnification- 100×). (C) Fluorescence microscopy for LC3 localization in cells treated with DMSO or 5 µM BITC for 6 h (9 h for MCF-7 cells; objective magnification- 100×). Except for transmission electron microscopy, each experiment was repeated at least twice with similar results.

**Figure 2:**
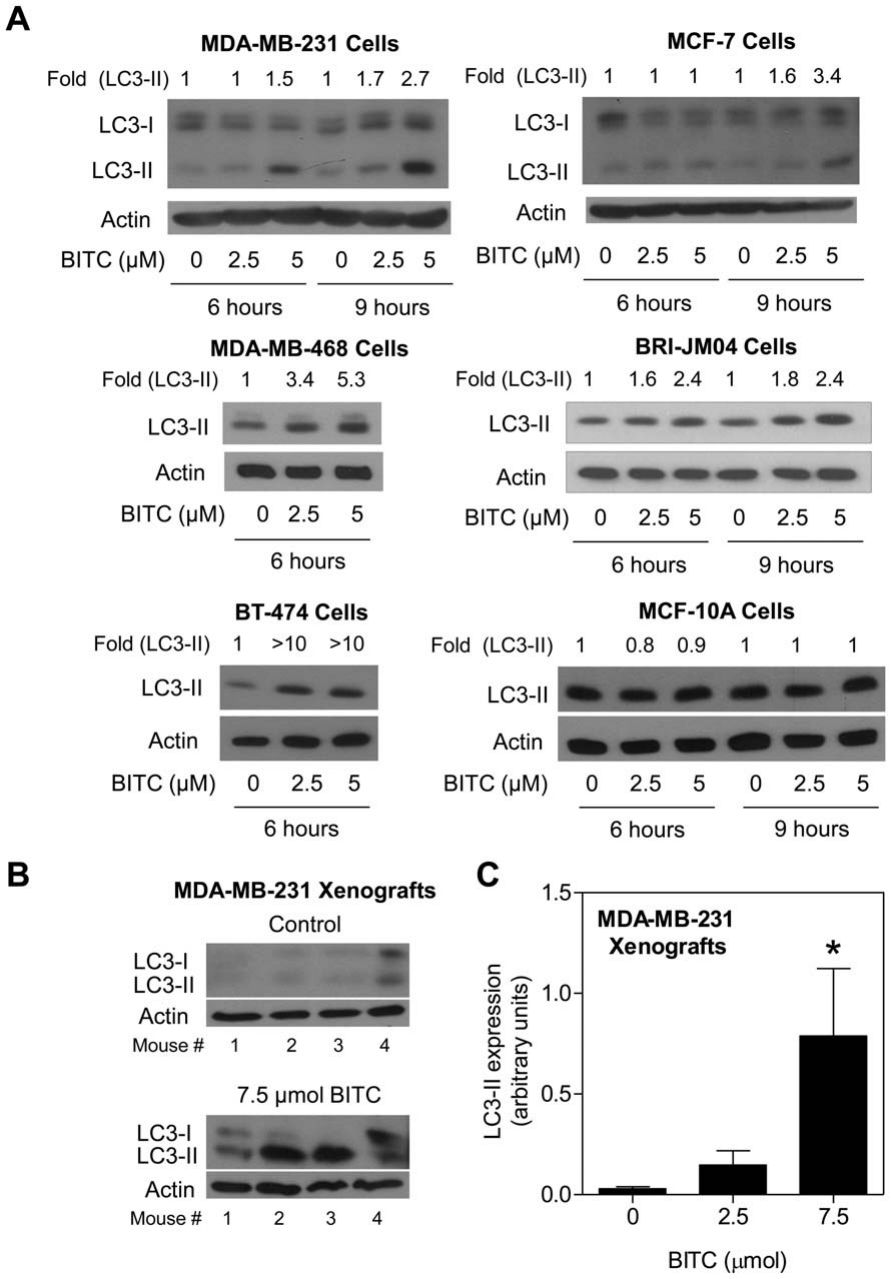
BITC treatment results in cleavage of LC3 in breast cancer cells and MDA-MB-231 xenografts. (A) Immunoblotting for full-length (LC3-I) and cleaved LC3 (LC3-II) using lysates from cells treated for specified time points with DMSO (control) or the indicated concentrations of BITC. Densitometric quantitation for cleaved LC3 (LC-II) relative to corresponding DMSO-treated control is shown on top of the bands. Similar results were obtained in two experiments using independently prepared lysates. (B) Immunoblotting for LC3-I and LC3-II using supernatants from MDA-MB-231 xenografts harvested from control and 2.5 or 7.5 µmol BITC-treated mice. Tumor tissues from 4 different mice of each group were used for immunoblotting. (C) Densitometric quantitation for cleaved LC3-II (arbitrary units). Results shown are mean ± SD (*n* = 4). *Significantly different (*P*<0.05) by one-way ANOVA with Dunnett's adjustment.

Fig. 1A depicts representative transmission electron microscopic images of MDA-MB-231 cells following 12 h treatment with dimethyl sulfoxide (DMSO; control) or BITC (2.5 µM). DMSO-treated control MDA-MB-231cells mostly exhibited healthy looking mitochondria. On the other hand, exposure of MDA-MB-231 cells to 2.5 µM BITC for 6 h (results not shown) or 12 h (Fig. 1A) resulted in appearance of double-membrane vacuoles resembling autophagosomes (identified by red arrows in Fig. 1A), which were infrequent in DMSO-treated control cells. Autophagic response to BITC treatment was confirmed by analysis of acidic vesicular organelles (AVOs) and cleavage of microtubule-associated protein 1 light chain 3 (LC3), which are hallmarks of autophagy [19]–[21]. The AVOs were visualized by fluorescence microscopy following staining with the lysosomotropic agent acridine orange. Acridine orange is a weak base that is able to move freely across biological membranes characterized by weak and diffuse green fluorescence. The protonated form of acridine orange accumulates in acidic compartments (lysosomes) and forms aggregates, which is characterized by yellow-orange fluorescence. Treatment of cells with BITC for 6 h (MDA-MB-231) or 9 h (MCF-7) resulted in formation of yellow-orange AVOs, which were rare in DMSO-treated control cells (Fig. 1B). Cleavage of LC3, a protein critical for autophagic machinery [19], is another widely used criterion for detection of autophagy. During autophagy LC3 (18 kDa) is cleaved to a 16 kDa intermediate (referred to as LC3-II) that localizes to the autophagosomes [20], [21]. Cleavage and recruitment of LC3-II to autophagosomes is characterized by punctate staining [21]. Staining for LC3 was diffuse in DMSO-treated control MDA-MB-231 and MCF-7 cells (Fig. 1C). The MDA-MB-231 and MCF-7 cells treated with BITC displayed punctate pattern of LC3 staining (Fig. 1C). To confirm the autophagic response to BITC, a panel of breast cell lines including MDA-MB-468 (ER negative, mutant p53), and BT-474 (ER positive, mutant p53), BRI-JM04 (ER negative), and a normal human mammary epithelial cell line (MCF-10A) were used. As seen in Fig. 1B, AVOs were also observed in MDA-MB-468, BT-474, and BRI-JM04 cells exposed to 5 µM BITC for 6 h. However, BITC-induced AVOs were not evident in MCF-10A normal human breast cell line. In addition, BITC exposure displayed punctate pattern of LC3-II staining in MDA-MB-468, BT-474, and BRI-JM04 breast cancer cells, but not in the MCF-10A cell line (Fig. 1C). Consistent with these results, cleavage of LC3 on treatment with BITC was also clearly visible in all five breast cancer cell lines (MDA-MB-231, MCF-7, MDA-MB-468, BT-474, and BRI-JM04), but not in the normal MCF-10A cells (Fig. 2A).

We have demonstrated previously that BITC treatment significantly retards growth of MDA-MB-231 xenografts in female athymic mice without causing any side effects [10]. We used archived tumor tissues from the same experiment to determine whether BITC treatment caused cleavage of LC3 *in vivo*. As shown in Fig. 2B, expression of full-length as well as cleaved LC3 was barely detectable in the MDA-MB-231 xenografts from control mice. On the other hand, the MDA-MB-231 tumors from the BITC-treated mice exhibited induction as well as cleavage of LC3 (Fig. 2B). Quantitatively, protein level of cleaved LC3 (LC3-II) was increased by about 26-fold in tumors from 7.5 µmol BITC-treated mice in comparison with those from control mice (*P*<0.05 by one-way ANOVA with Dunnett's adjustment; Fig. 2C).

**Figure 3:**
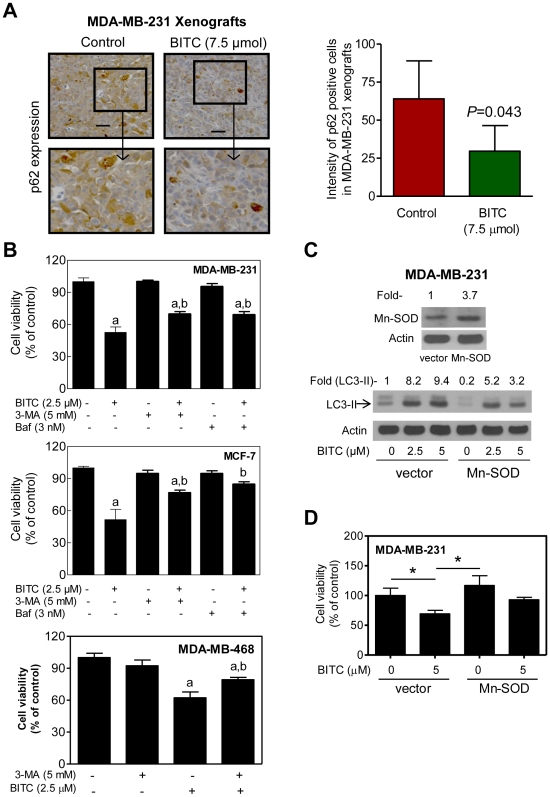
BITC-mediated growth inhibition is attenuated by autophagy inhibitors. (A) Immunohistochemical analysis for p62 expression in MDA-MB-231 xenograft from a representative mouse of the control group and a mouse of the 7.5 µmol BITC treatment group. Enlarged images for identified area are shown below. Quantitation of p62 expression is shown in the right hand panel. Six and four tumor sections, respectively, from mice of control and 7.5 µmol BITC treatment groups were analyzed for p62 expression. Difference in p62 expression between control and 7.5 µmol BITC groups was statistically significant (*P* = 0.043) by two-tailed Student's *t*-test. Scale bar- 40 µm; 200× magnification. (B) Viability of MDA-MB-231, MCF-7, MDA-MB-468 cells following 6 h treatment with 2.5 µM BITC in the absence or presence of 5 mM 3-MA or 3 nM Baf (2 h pre-treatment). Results shown are mean ± SD (*n* = 3). Significantly different (*P*<0.05) compared with ^a^DMSO-treated control and ^b^BITC alone treatment group by one-way ANOVA followed by Tukey's multiple comparison test (for MDA-MB-231 and MCF-7 cells) or Bonferroni's multiple comparison test (for MDA-MB-468 cells). (C) Immunoblotting for Mn-SOD and LC3 using lysates from MDA-MB-231 cells stably transfected with the empty pcDNA3.1 vector or the same vector encoding for Mn-SOD and treated for 6 h with DMSO or BITC (2.5 and 5 µM). Numbers above the bands represent densitometric quantitation relative to empty vector-transfected cells treated with DMSO (lane 1). (D) Viability of empty vector-transfected control and Mn-SOD overexpressing MDA-MB-231 cells following 6 h treatment with DMSO or 5 µM BITC. Results shown are mean ± SD (*n* = 3). *Significantly different (*P*<0.05) between the indicated groups by one-way ANOVA followed by Bonferroni's multiple comparison test. Each experiment was repeated and representative data from one such experiment are shown.

Additional evidence for *in vivo* autophagy was obtained by immunohistochemical analysis of p62 (also known as p62/SQSTM1 or sequestosome 1). The p62 protein not only interacts with LC3 but also accumulates in mouse models of defective autophagy [22], [23]. Immunohistochemical staining for p62 is shown in Fig. 3A (*left panel*). As seen in Fig. 3A (*right panel*), p62 expression was quantitatively decreased by about 54% in the MDA-MB-231 tumors from BITC-treated mice compared with those from control mice (*P* = 0.043 by two-tailed Student's *t*-test). Collectively, these results provided evidence for BITC-induced autophagy in cultured and xenografted human breast cancer cells.

### Pharmacologic Suppression of Autophagy Attenuated Growth Inhibitory Effect of BITC

Next, we designed experiments to determine the functional significance of autophagy in BITC-mediated suppression of cell viability using a pair of chemical inhibitors of autophagy, 3-methyl adenine (3-MA) and bafilomycin A1 (Baf). For these experiments, the cells were first pre-treated for 2 h with 5 mM 3-MA or 3 nM Baf and then exposed to 2.5 µM BITC for 6 h in the presence of the inhibitor prior to trypan blue dye exclusion assay. The inhibitor concentrations were optimized based on lack of cellular toxicity and inhibition of BITC-mediated cleavage of LC3 (results not shown). Viability of MDA-MB-231, MCF-7, and MDA-MB-468 cells was decreased by about 38–48% upon 6 h treatment with 2.5 µM BITC (^a^ *P*<0.05 compared with DMSO-treated control by one-way ANOVA with Tukey's or Bonferroni's adjustment; Fig. 3B). While 3-MA alone (an inhibitor of phosphatidylinositol 3-phosphate kinase) was non-toxic to these cells, suppression of cell viability resulting from BITC exposure was partially but statistically significantly attenuated in the presence of 3-MA (^b^ *P*<0.05 between BITC alone group and BITC+3-MA group, Fig. 3B). Similarly a non-toxic concentration of Baf (an inhibitor of vacuolar type H^+^-ATPase) conferred partial yet significant protection against BITC-mediated growth inhibition in both MDA-MB-231 and MCF-7 cells (Fig. 3B). Because BITC treatment also causes apoptotic cell death in these breast cancer cells [9], [14], partial protection against cell killing is expected. Thus, it is reasonable to conclude that autophagy induction by BITC did not impart a survival advantage in breast cancer cells.

### BITC-Induced Autophagy Was Partially Attenuated by Superoxide Dismutase Overexpression

We have shown previously that the BITC-induced apoptotic cell death in human breast cancer cells is accompanied by ROS production [14]. Moreover, the BITC-induced ROS production and apoptosis are significantly reversed by overexpression of catalase or superoxide dismutase (SOD) [14]. We raised the question of whether BITC-induced autophagy in our model was dependent on ROS production, which was likely based on recent literature implicating ROS in autophagy induction [17]. The MDA-MB-231 cells with stable overexpression of manganese SOD (Mn-SOD) were partially resistant to BITC-mediated (6 h treatment) cleavage of LC3 compared with empty vector-transfected control cells (Fig. 3C). The LC3 punctate staining resulting from BITC exposure was also partially attenuated by Mn-SOD overexpression (results not shown). Mn-SOD overexpressing MDA-MB-231 cells were relatively more resistant to killing by BITC exposure compared with empty vector-transfected control (31% decrease in cell viability in empty vector-transfected cells in comparison with DMSO-treated control *versus* 21% decrease in cell viability in Mn-SOD overexpressing cells in comparison with respective DMSO-treated control), but the difference did not reach statistical significance (Fig. 3D). On the other hand, BITC-mediated increase in histone-associated DNA fragment release into the cytosol (a measure of apoptotic cell death) was completely abolished in the Mn-SOD overexpressing cells in comparison with empty vector transfected cells (results not shown). These results indicated that the ROS production alone did not fully account for the BITC-induced autophagy in MDA-MB-231 cells. Nevertheless, it was obvious that BITC treatment simultaneously triggered both apoptotic and autophagic cell death in breast cancer cells.

### BITC Treatment Caused Suppression of mTOR Activity

**Figure 4:**
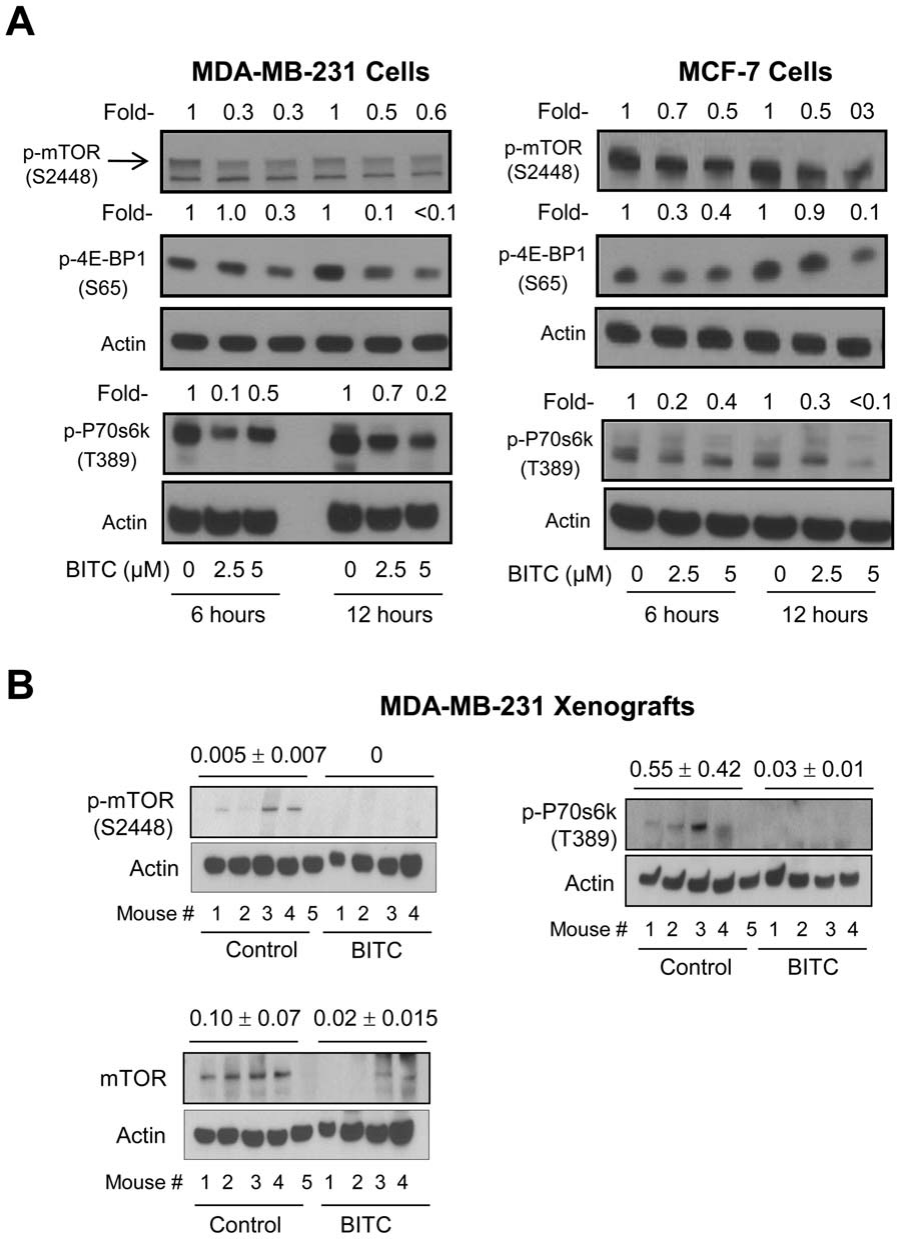
BITC treatment suppresses phosphorylation of mTOR and its targets in MDA-MB-231 and MCF-7 cells. (A) Immunoblotting for phospho-(S2448)-mTOR, phospho-(S65)-4E-BP1, and phospho-(T389)-P70s6k using lysates from MDA-MB-231 and MCF-7 cells treated with DMSO or BITC (2.5 or 5 µM BITC) for 6 or 12 h. Numbers on top of the bands represent change in protein level relative to corresponding DMSO-treated control. Immunoblotting for each protein was performed at least twice using independently prepared lysates and the results were comparable. (B) Immunoblotting for phospho-(S2448)-mTOR, total mTOR, and phospho-(T389)-P70s6k using supernatant from MDA-MB-231 xenografts harvested from control mice (*n* = 5) and 7.5 µmol BITC-treated mice (*n* = 4). Densitometric quantitation (mean arbitrary units ± SD) for control and BITC-treated groups is shown on top of the bands. Difference in expression of mTOR and phospho-P70s6k between control and BITC groups was statistically significant by two-tailed Student's *t*-test.

Serine-threonine kinase mTOR has emerged as a key negative regulator of autophagy in cancer cells [20]. Initially we determined the effect of BITC treatment on phosphorylation of mTOR as well as its downstream substrates P70s6k and 4E-BP1. As shown in Fig. 4A, BITC-treated MDA-MB-231 and MCF-7 cells exhibited a marked decrease in levels of phospho-(S2448)-mTOR, phospho-(S65)-4E-BP1, and phospho-(T389)-P70s6k. In addition, levels of phospho-(S2448)-mTOR, total mTOR, and phospho-(T389)-P70s6k were decreased by 100%, 80% (*P* = 0.051 by two-tailed Student's *t*-test), and 95% (*P* = 0.046 by two-tailed Student's *t*-test), respectively, in MDA-MB-231 xenografts from 7.5 µmol BITC-treated mice compared with those from control mice (Fig. 4B). These results demonstrated inhibition of mTOR by BITC *in vitro* and *in vivo*.

**Figure 5:**
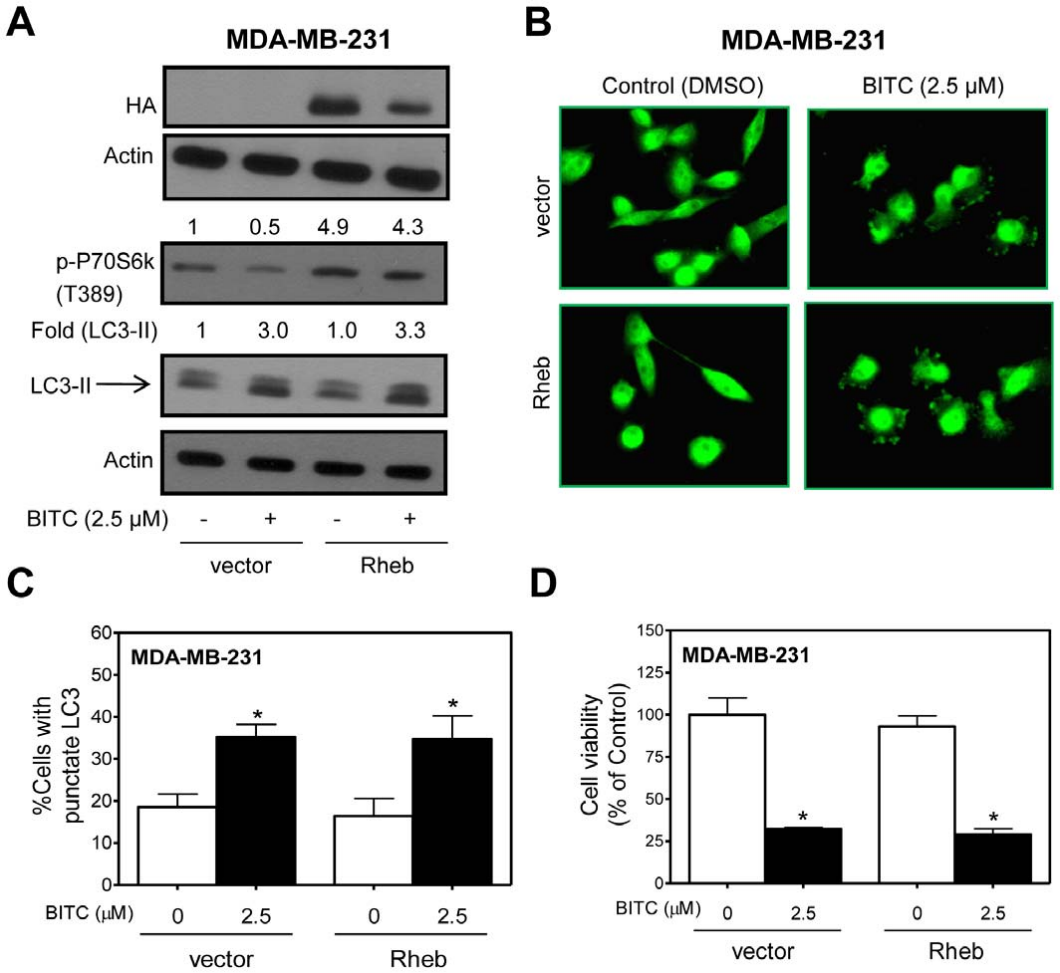
Role of mTOR suppression in BITC-induced autophagy. (A) Immunoblotting for HA-tag, phospho-(T389)-P70s6k, and LC3 using lysates from MDA-MB-231 cells transiently transfected with the empty vector or vector encoding for HA-Rheb and treated with DMSO (control) or 2.5 µM BITC for 6 h. Densitometric quantitation relative to empty vector-transfected cells treated with DMSO is shown on top of the bands. (B) Microscopic visualization of LC3 immunostaining in empty vector transfected control cells and HA-Rheb transfected MDA-MB-231 cells following 6 h treatment with DMSO or 2.5 µM BITC (×100 objective magnification). (C) Percentage of cells with punctate LC3 in MDA-MB-231 cells transiently transfected with the empty vector or vector encoding for HA-Rheb and treated with DMSO (control) or 2.5 µM BITC for 6 h. (D) Viability in MDA-MB-231 cells transiently transfected with the empty vector or vector encoding for HA-Rheb and treated with DMSO (control) or 2.5 µM BITC for 6 h. Results shown are mean ± SD (*n* = 3). *Significantly different (*P*<0.05) compared with corresponding DMSO-treated control by one-way ANOVA followed by Bonferroni's multiple comparison test. Each experiment was repeated twice and the results were comparable.

We next conducted experiments involving transient overexpression of Rheb to determine the functional significance of mTOR suppression in autophagic response to BITC. The Rheb activates mTOR by antagonizing its endogenous inhibitor FKBP38 [24]. Ectopic expression of Rheb in MDA-MB-231 cells resulted in activation of mTOR as evidenced by about 4.9-fold increase in T389 phosphorylation of P70s6k (Fig. 5A). However, Rheb-mediated activation of mTOR did not impart a meaningful protection against BITC-induced cleavage of LC3 (Fig. 5A), LC3 puncta (Fig. 5B,C) or cell growth inhibition (Fig. 5D). These observations were not unique to the MDA-MB-231 cell line because Rheb-mediated activation of mTOR failed to confer any protection against BITC-induced autophagy or growth inhibition in the MCF-7 cell line (results not shown). These results indicated that inhibition of mTOR was not a contributing mechanism in execution of BITC-induced autophagy.

### BITC Treatment Resulted in Increased Acetylation of FoxO1

**Figure 6:**
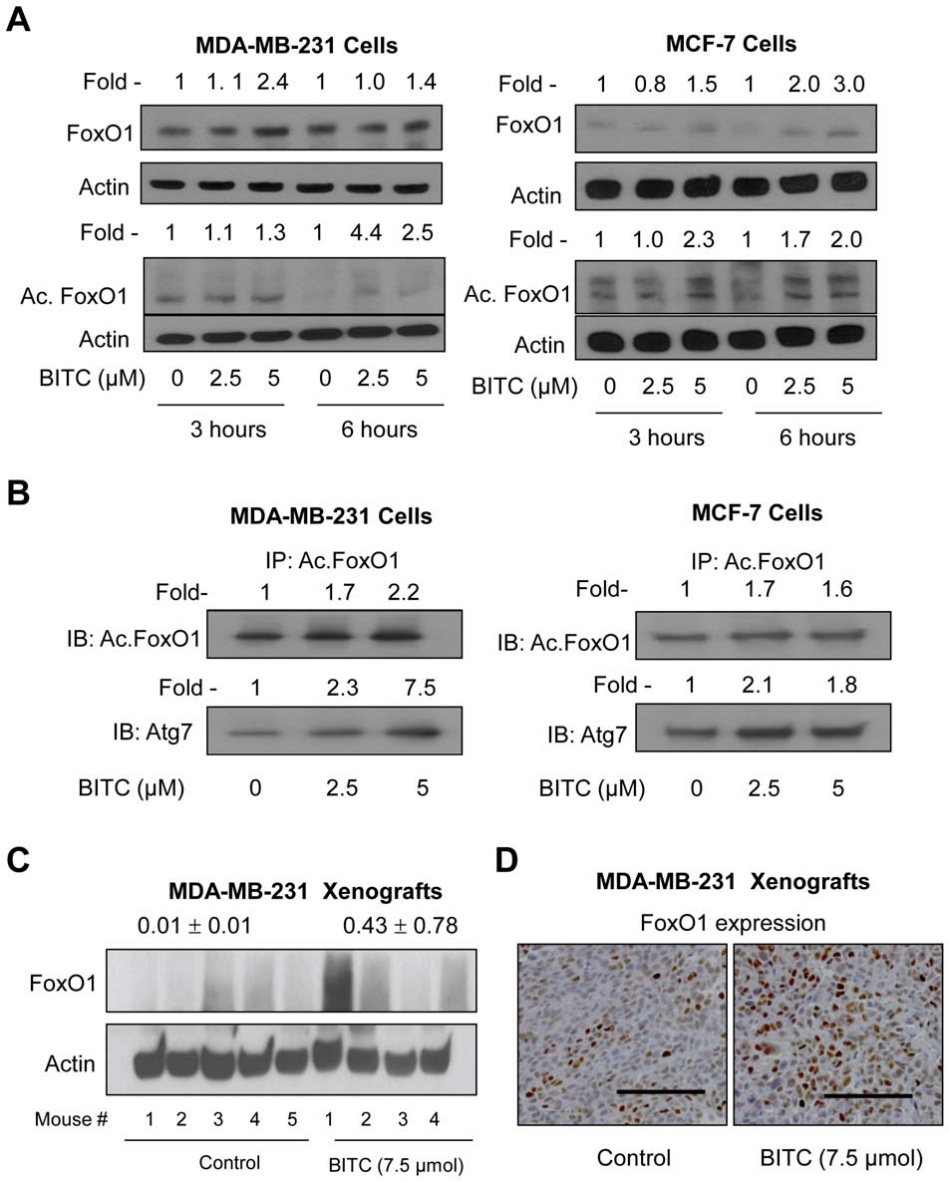
BITC treatment causes acetylation of FoxO1 in MDA-MB-231 and MCF-7 cells. (A) Immunoblotting for FoxO1 and acetylated FoxO1 (abbreviated as Ac.FoxO1) using lysates from MDA-MB-231 and MCF-7 cells treated with DMSO (control) or 2.5 and 5 µM BITC for the indicated time periods. Densitometric quantitation relative to corresponding DMSO-treated control is shown on top of the bands. Similar results were obtained in two experiments using independently prepared lysates. (B) Immunoblotting for Ac.FoxO1 and Atg7 using immunoprecipitates (using anti-Ac.FoxO1 antibody) from the MDA-MB-231 and MCF-7 cells treated for 6 h with DMSO (control) or BITC (2.5 or 5 µM). (C) Immunoblotting for FoxO1 using supernatant from MDA-MB-231 xenografts harvested from control mice (*n* = 5) and 7.5 µmol BITC-treated mice (*n* = 4). Densitometric quantitation (mean arbitrary units ± SD) for control and BITC treatment groups is shown on top of the bands. (D) Immunohistochemical analysis for FoxO1 expression in MDA-MB-231 xenograft from a representative mouse of the control group and a mouse of the 7.5 µmol BITC group. Seven and six tumor sections, respectively, from the mice of control and 7.5 µmol BITC treatment groups were analyzed for immunohistochemical analysis of FoxO1. Scale bar- 100 µm (200× magnification).

FoxO1 was recently identified as a critical regulator of autophagy induction by H_2_O_2_ and serum starvation [25]. This study also showed that autophagy was associated with acetylation of FoxO1 leading to its increased interaction with Atg7 [25]. We proceeded to test possible involvement of FoxO1 in autophagy induction in our model. The level of total and acetylated FoxO1 was increased modestly in BITC-treated MDA-MB-231 and MCF-7 cells (Fig. 6A). In addition, the BITC treatment resulted in increased interaction between acetylated FoxO1 and Atg7 as revealed by an experiment involving immunoprecipitation of acetylated FoxO1 followed by immunoblotting with the use of anti-Atg7 antibody (Fig. 6B). The BITC-mediated increase in total FoxO1 protein level was evident in MDA-MB-231 xenografts in Western blotting (Fig. 6C) as well as immunohistochemical experiments (Fig. 6D). These results indicated induction and increased acetylation of FoxO1 in breast cancer cells upon treatment with BITC.

### Knockdown of FoxO1 Protein Conferred Protection Against BITC-Induced Autophagy

**Figure 7:**
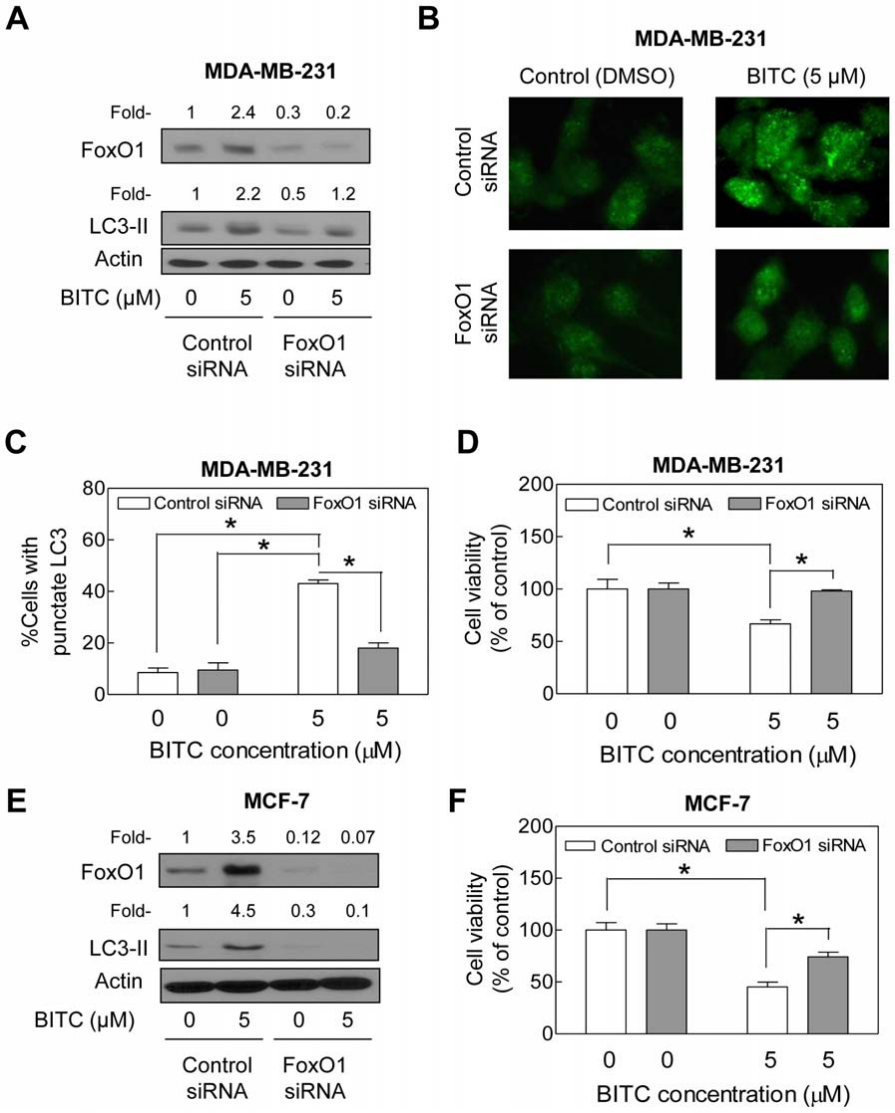
FoxO1 is essential for BITC-induced autophagy in MDA-MB-231 and MCF-7 cells. (A) Immunoblotting for FoxO1 and LC3 using lysates from MDA-MB-231 cells transiently transfected with a nonspecific siRNA or the FoxO1-targeted siRNA and treated for 6 h with DMSO or 5 µM BITC. Numbers on top of the bands represent change in protein level relative to DMSO-treated nonspecific siRNA-transfected cells. (B) Immunofluorescence microscopy for LC3 in MDA-MB-231 cells transiently transfected with a nonspecific siRNA or the FoxO1-targeted siRNA and treated for 6 h with DMSO or 5 µM BITC. (C) Quantitation of LC3 puncta and (D) cell viability in MDA-MB-231 cells transiently transfected with a nonspecific siRNA or the FoxO1-targeted siRNA and treated for 6 h with DMSO or 5 µM BITC. (E) Immunoblotting for FoxO1 and LC3 using lysates from MCF-7 cells transiently transfected with a nonspecific siRNA or the FoxO1-targeted siRNA and treated for 6 h with DMSO or 5 µM BITC. Numbers on top of the immunoreactive bands represent change in protein level relative to DMSO-treated nonspecific siRNA-transfected cells. (F) Viability of MCF-7 cells transiently transfected with a nonspecific siRNA or the FoxO1-targeted siRNA and treated for 6 h with DMSO or 5 µM BITC. Results in panels C, D, and F are mean ± SD (*n* = 3). Significantly different (*P<0.05) between the indicated groups by one-way ANOVA followed by Bonferroni's test. Each experiment was done twice with similar results, and representative data from one such experiment are shown.

Level of FoxO1 protein was decreased by about 70% in MDA-MB-231 cells transiently transfected with the FoxO1-targeted siRNA in comparison with nonspecific siRNA-transfected cells (Fig. 7A). Induction of FoxO1 protein was completely abolished by RNA interference of FoxO1 (Fig. 7A). The BITC-mediated cleavage of LC3 (Fig. 7A) and LC3 puncta (Fig. 7B,C) were clearly evident in nonspecific siRNA-transfected MDA-MB-231 cells but not in the cells transfected with FoxO1-specific siRNA. In agreement with these results, RNA interference of FoxO1 conferred significant protection against BITC-mediated growth inhibition in MDA-MB-231 cells (Fig. 7D). Level of FoxO1 protein was decreased by about 88% in MCF-7 cells transfected with the FoxO1-targeted siRNA (Fig. 7E). RNA interference of FoxO1 conferred significant protection against BITC-induced cleavage of LC3 compared with nonspecific siRNA-transfected MCF-7 cells (Fig. 7E). In addition, the MCF-7 cell line with knockdown of FoxO1 protein was significantly more resistant to BITC-mediated growth inhibition compared with corresponding nonspecific siRNA-transfected cells (Fig. 7F). Together these results pointed towards critical involvement of FoxO1 in regulation of BITC-induced autophagy in both MDA-MB-231 and MCF-7 cells.

## Discussion

The present study shows that BITC treatment causes autophagy in breast cancer cells that is not influenced by the estrogen receptor expression. Recent studies point towards an important role of cytoplasmic p53 in regulation of autophagy [26]. The p53 status has no obvious impact on BITC-induced autophagy because this response is observed in both MCF-7 (wild-type p53) and MDA-MB-231 cells (mutant p53). Autophagic response to BITC is not restricted to cultured breast cancer cells as MDA-MB-231 xenografts from the BITC-treated mice exhibit features characteristic of autophagy, including cleavage of LC3 and suppression of p62 expression. Autophagy induction by some stimuli contributes to drug resistance in cancer cells [18], [27]. For example, inhibition of autophagy by chloroquine augments activity of the alkylating agent cyclophosphamide in a *Myc*-driven model of lymphoma [27]. We have also shown previously that D,L-sulforaphane, a synthetic racemic analogue of broccoli constituent L-sulforaphane, causes autophagy that serves to prevent cytosolic release of cytochrome *c* and apoptosis in PC-3 and LNCaP human prostate cancer cells [28]. The present study reveals that autophagy is not a cytoprotective mechanism against growth inhibition by BITC in breast cancer cells.

Evidence continues to accumulate to suggest that autophagic response to some anti-cancer agents is mediated by ROS, including arsenic trioxide [29], [30]. Arsenic trioxide causes apoptotic and/or autophagic death in cancer cells, and the cytotoxicity of this agent is dependent on ROS production [29]. ROS-dependent autophagy induction by 2-methoxyestradiol, an investigational anti-cancer agent currently in clinical trials, has also been demonstrated [31]. Mechanism by which ROS regulate autophagy is not fully understood, but up-regulation of Beclin 1, Atg4 oxidation, and mitochondrial dysfunction are some possibilities explaining this relationship [32], [33]. We have shown previously that BITC treatment inhibits complex III of the mitochondrial respiratory chain leading to ROS production in MDA-MB-231 and MCF-7 cells [14]. Furthermore, prooxidant as well as proapoptotic effects of BITC in these cells are significantly attenuated by ectopic expression of catalase and SOD [14]. The present study reveals that autophagic response to BITC in breast cancer cells is partially dependent on ROS production. Suppression of ROS production through stable overexpression of Mn-SOD confers partial protection against BITC-induced autophagy. However, additional work is needed to test whether ROS-dependent autophagy by BITC involves upregulation of Beclin 1, Atg4 oxidation, or any other mechanism.

Initially we focused on mTOR, which negatively regulates autophagy in yeast and mammals [34], to gain insight into the mechanism of BITC-induced autophagy. Even though BITC treatment inhibits mTOR activity as evidenced by a reduction in phosphorylation of its downstream targets (P70s6k and 4E-BP1), autophagic response to BITC is not attenuated by activation of mTOR through overexpression of its positive regulator Rheb in either MDA-MB-231 or MCF-7 cells. To our surprise, Rheb-mediated activation of mTOR fails to confer any protection against cell killing or apoptotic DNA fragmentation by BITC (Singh SV, unpublished observations) in either MDA-MB-231 or MCF-7 cells. These observations are intriguing because of established role of mTOR in regulation of cell cycle, apoptosis, protein translation, and ribosomal biogenesis [34], [35].

FoxO1, a member of the forkhead O family of transcription factors, is considered a tumor suppressor because of its ability to cause cell cycle arrest and DNA repair [36]. Using serum-starved (24 h) or H_2_O_2_-treated (0.5 mM, 6 h) H1299 human lung cancer and HCT-116 human colon cancer cells as experimental models, FoxO1 was identified as a mediator of autophagy [25]. FoxO1-mediated autophagy was shown to be independent of its transcriptional activity but accompanied by acetylation leading to increased binding with Atg7 [25]. We reasoned that autophagic response to BITC might be mediated by FoxO1. Indeed, we observed that BITC treatment causes modest induction as well as increased acetylation of FoxO1 in both MDA-MB-231 and MCF-7 cells. Moreover, the BITC-mediated acetylation of FoxO1 promotes its interaction with Atg7 in both cells as evidenced by immunoprecipitation-immunoblotting assay. Because BITC-induced autophagy as well as cell growth inhibition is significantly attenuated by knockdown of FoxO1, we conclude that this protein plays a critical role in autophagic cell death by BITC treatment in human breast cancer cells.

In conclusion, the present study shows that BITC treatment causes FoxO1-mediated autophagic cell death in breast cancer cells, which is neither a cell-line specific phenomenon nor affected by the estrogen receptor or p53 status.

## Methods

### Ethics Statement

We used archived tumor tissues from our previously published xenograft study [10] to obtain *in vivo* evidence for BITC-induced autophagy. Use of mice and their care for this study [10] was approved by the University of Pittsburgh Institutional Animal Care and Use Committee (IACUC; protocol number 1004983-4).

### Reagents

BITC (purity- about 98%) and Baf were purchased from LKT Laboratories (St. Paul, MN). Cell culture reagents, fetal bovine serum, and OligoFECTAMINE were purchased from Invitrogen-Life Technologies (Carlsbad, CA). The 3-MA and acridine orange were purchased from Sigma-Aldrich (St. Louis, MO). Fugene6 and a kit for quantification of histone-associated DNA fragment release into the cytosol were purchased from Roche Diagnostics (Indianapolis, IN). Antibodies were purchased from the following sources- anti-LC3 antibody recognizing both full-length and cleaved forms was from MBL International (Woburn, MA); antibodies against p62 (for immunoblotting), phospho-(S2448)-mTOR, phospho-(T389)-P70s6k, phospho-(S65)-4E-BP1, FoxO1 (immunoblotting and immunohistochemistry), Atg7, and LC3 isoform B (LC3B) were from Cell Signaling Technology (Danvers, MA); anti-acetylated FoxO1 and anti-HA antibodies were from Santa Cruz Biotechnology (Santa Cruz, CA); anti-actin antibody was purchased from Sigma-Aldrich; and anti-mTOR and anti-Mn-SOD antibodies were from EMD Chemicals-Calbiochem (Gibbstown, NJ). An antibody against mTOR used for immunoblotting using MDA-MB-231 xenograft supernatants was from Cell Signaling Technology.

### Cell Lines and Cell Viability Assay

The MDA-MB-231, MCF-7, MDA-MB-468, BT-474, and MCF-10A cell lines were purchased from the American Type Culture Collection (Manassas, VA) and maintained by following the supplier's recommendations. The BRI-JM04 cells, derived from spontaneously developing mammary tumor of a MMTV-*neu* mouse were generously provided by Dr. Anne Lenferink (Biotechnology Research Institute, Montreal, Canada). Each cell line was maintained at 37°C in an atmosphere of 95% air and 5% CO_2_. Stock solution of BITC was prepared in DMSO and an equal volume of DMSO (final concentration 0.1%) was added to the controls. Effect of BITC on cell viability was determined by trypan blue dye exclusion assay [37].

### Transmission Electron Microscopy

The MDA-MB-231 cells (2×10^5^) were seeded in six-well plates and allowed to attach by overnight incubation. The cells were then treated with either DMSO (control) or 2.5 µM BITC for 6 or 12 h at 37°C. Cells were fixed in ice-cold 2.5% electron microscopy grade glutaraldehyde in phosphate-buffered saline (PBS) overnight at 4°C. Fixed samples were washed three times in PBS, and then post-fixed in 1% osmium tetroxide containing 0.1% potassium ferricyanide for 1 h. Following three PBS washes, samples were dehydrated through a graded series of 30% to100% ethanol. Propylene oxide (100%) was then infiltrated in a 1∶1 mixture of propylene oxide and Polybed 812 epoxy resin (Epon) (Polysciences, Warrington, PA) for 1 h. After several changes of 100% resin over 24 h, samples were embedded in molds, cured at 37°C overnight, followed by additional hardening at 65°C for 2 days. Ultrathin (60 nm) sections were collected, and stained with 2% uranyl acetate in 50% methanol for 10 min followed by staining with 1% lead citrate for 7 min. Sections were imaged using JEOL JEM 1011 transmission electron microscope (Peabody, MA).

### Detection of Acidic Vesicular Organelles (AVOs)

The MDA-MB-231, MCF-7, MDA-MB-468, BT-474, BRI-JM04, and MCF-10A cells (1×10^5^) were seeded on coverslips in 12-well plates and allowed to attach by overnight incubation. Following treatment with DMSO (control) or BITC (5 µM) for 6 or 9 h, the cells were stained with 1 µg/mL acridine orange in PBS for 15 min, washed with PBS, and examined under a Leica fluorescence microscope (Leica Microsystems, Bannockburn, IL) at 100× objective magnification.

### Immunofluorescence Microscopy

The MDA-MB-231, MCF-7, MDA-MB-468, BT-474, BRI-JM04, and MCF-10A cells (1×10^5^) were grown on coverslips in 12 well-plates and allowed to attach. The cells were then exposed to the desired concentration of BITC or DMSO (control) for 6 or 9 h at 37°C. The cells were washed with PBS and fixed in 2% paraformaldehyde overnight at 4°C. The cells were permeabilized with 0.1% Triton X-100 for 15 min at room temperature, washed with PBS, and blocked with PBS containing 0.5% bovine serum albumin and 0.15% glycine for 1 h at room temperature. Following incubation with anti-LC3 antibody, cells were treated with 2 µg/mL of Alexa Fluor 488 conjugated secondary antibody for 1 h at room temperature. Ppunctate pattern of LC3 was visualized under a microscope at 100× objective magnification. Cells with punctate LC3 were counted for each treatment group.

### Western Blotting

Lysates from BITC-treated and DMSO-treated cells and supernatants from MDA-MB-231 tumor xenografts were prepared as described by us previously [38], [39]. Lysate proteins were resolved by 6–12.5% sodium dodecyl sulfate polyacrylamide gel electrophoresis and transferred onto polyvinylidene fluoride membrane. Immunoblotting was performed as described by us previously [38], [39]. Change in protein expression was determined by densitometric scanning using UN-SCAN-IT software version 5.1 (Silk Scientific Corporation, Orem, Utah).

### Immunohistochemical Analysis for p62 and FoxO1 in MDA-MB-231 Xenografts

Immunohistochemistry was performed as previously described for other proteins [39]. Briefly, MDA-MB-231 tumor sections (4–5 µm) were quenched with 3% hydrogen peroxide and blocked with normal serum. The sections were then probed with the desired primary antibody (anti-p62 or anti-FoxO1), washed with Tris-buffered saline, and incubated with an appropriate secondary antibody. The characteristic brown color was developed by incubation with 3,3′-diaminobenzidine. The sections were counterstained with Mayer's Hematoxylin (Sigma-Aldrich) and examined under a Leica microscope. The images were captured and analyzed with Image ProPlus 5.0 software (Media Cybernetics, Bethesda, MD) as previously described [39]. At least four non-overlapping images were captured and analyzed with the Red-Green-Blue color histogram tool from Image ProPlus 5.0 software.

### Stable Overexpression of Mn-Superoxide Dismutase (Mn-SOD)

MDA-MB-231 cells were stably transfected with empty pcDNA3.1 vector or pcDNA3.1 vector encoding for Mn-SOD (8 µg plasmid DNA) using Fugene6. The cells were selected by culture in medium supplemented with 800 µg/mL G-418 over 2 months. Mn-SOD overexpression in stably transfected cells was confirmed by immunoblotting. The cells were processed for LC3 immunoblotting, trypan blue dye exclusion assay, and analysis of histone-associated DNA fragment release into the cytosol, which is a well-accepted technique for quantitation of apoptotic cell death. Histone-associated DNA fragment release into the cytosol was determined using Cell Death ELISA Detection kit from Roche Diagnostics-USA (Indianapolis, IN) according to the manufacturer's instructions.

### Overexpression of Rheb

The MDA-MB-231 and MCF-7 cells (2×10^5^) were plated in 6-well plates, allowed to attach overnight, and transiently transfected with 2–4 µg of pcDNA3.1 vector encoding for HA-Rheb or empty vector using Fugene6 transfection regent. After 24 h, the cells were treated with DMSO (control) or 2.5 µM BITC for 6 h. The cells were then processed for immunoblotting (HA tag, phospho-P70s6k or LC3), immunofluorescence microscopy for LC3, and trypan blue dye exclusion assay.

### Immunoprecipitation-Immunoblotting Assay for FoxO1-Atg7 Interaction

Aliquots containing 200 µg of total lysate protein from cells treated for 6 h with DMSO (control) or BITC (2.5 or 5 µM) were incubated overnight at 4°C with 4 µg of anti-acetylated FoxO1 antibody. Protein G-agarose beads (50 µL; Santa Cruz Biotechnology) were then added to each sample and the incubation was continued for an additional 2 h at 4°C. Immunoprecipitates were washed five times with lysis buffer and subjected to sodium-dodecyl sulfate polyacrylamide gel electrophoresis followed by immunoblotting using anti-acetylated FoxO1 or anti-Atg7 antibody.

### RNA Interference of FoxO1

Cells (1×10^5^ cells in 6-well plates) were transfected at 30–50% confluency with either 200 nM FoxO1-targeted siRNA (Cell Signaling Technology) or a control non-specific siRNA (Qiagen, Valencia, CA). After 24 h, the cells were exposed to desired concentration of BITC or DMSO and the incubation was continued for an additional 6 h. Cells were then collected and processed for immunoblotting of FoxO1 and LC3, immunofluorescence microscopy for LC3 localization, and trypan blue dye exclusion assay.
